# Chemical Indices and Kinetic Evaluation of β-Sitosteryl Oleate Oxidation in a Model System of Bulk Oil

**DOI:** 10.3390/molecules27227833

**Published:** 2022-11-14

**Authors:** Manat Chaijan, Worawan Panpipat, Ling-Zhi Cheong

**Affiliations:** 1 Food Technology and Innovation Research Center of Excellence, School of Agricultural Technology and Food Industry, Walailak University, Nakhon Si Thammarat 80160, Thailand; 2 Zhejiang-Malaysia Joint Research Laboratory for Agricultural Product Processing and Nutrition, College of Food and Pharmaceutical Science, Ningbo University, Ningbo 315211, China

**Keywords:** phytosterol, phytosterol ester, lipid oxidation, stability

## Abstract

Concerns have been raised about the safety and tolerability of phytosterol esters due to their vulnerability to oxidation. Herein, oxidation of the unsaturated fatty acid-phytosterol ester, namely β-sitosteryl oleate, was observed in comparison to native β-sitosterol after accelerated storage at 65 °C for 35 days in a bulk oil model system. Depending on the sterol structure, various chemical indices of lipid oxidation, including hydroperoxide value (HPV), thiobarbituric acid reactive substances (TBARS), *p*-anisidine value (AnV), and 7-keto derivatives, changed at varying rates in both samples. Such indicators for β-sitosteryl oleate appeared to be obtained at higher concentrations than those for β-sitosterol. The first order kinetic was used to describe the losses of β-sitosteryl oleate and β-sitosterol in bulk oil. It was discovered that the β-sitosteryl oleate (k = 0.0202 day^−1^) underwent oxidative alteration more rapidly than β-sitosterol (k = 0.0099 day^−1^). Results indicated that physical structure was the principal factor in the determination of storage stability of phytosterol and its ester. Research on antioxidants and storage techniques can be expanded in order to reduce the oxidative loss of phytosterol esters during storage and improve the safety and tolerability of phytosterol esters.

## 1. Introduction

Due to their insoluble nature in both water and oil, phytosterols can only be added to foods in a restricted number of forms [1]. Consequently, phytosterols have been esterified using a variety of techniques to produce phytosterol esters, in order to facilitate preparations in fatty food systems. This was done to increase the industrial usage of phytosterols and to improve their bioaccessibility. Phytosterol esters, which have lower melting points and greater oil solubility, have shown similar physiological effects as phytosterols [2]. Phytosterol esters have received approval as cholesterol-lowering agents from the European Food Safety Authority (EFSA), Health Canada, and the United States Food and Drug Administration (USFDA). Functional foods containing phytosterol derivatives have become more common as a result [3]. Due to their ability to lower cholesterol and be fat-soluble, phytosterol esters have been incorporated as functional food ingredients in a variety of lipid-containing food products, such as emulsion-based foods, vegetable oils, and milk products, with excellent tolerance results and no negative side effects at daily intake [1,3,4,5,6,7]. According to Miettinen and Vanhanen [8], the food matrix had an impact on the cholesterol-reducing effect, and phytosterol esters were more successful at lowering plasma cholesterol than the original phytosterol preparations.

Foods containing lipids come in two main forms: a bulk lipid and a lipid dispersion in an aqueous matrix. In this way, phytosterol esters can be added to food systems based on large amounts of oil or emulsion [9]. Nevertheless, phytosterol esters can undergo oxidation much like other lipids, and this process might take diverse forms depending on the food matrix [10]. According to the free radical process, both phytosterol esters and free phytosterols undergo autooxidation [11]. Similar to how fatty acids are oxidized, so too are sterols. It has been shown that during the oxidation of phytosterol, sterol hydroperoxides primarily occur at the allylic C-7 location [11] as well as phytosterol oxidation products (POPs), which include keto-, hydroxy-, and epoxy-derivatives [12]. Research has documented evidence of secondary POPs’ negative consequences [13,14]. POPs have been shown to have pro-atherogenic and pro-inflammatory effects [3]. The pathophysiology of atherosclerosis involves dietary sterols and the byproducts of their oxidation [15]. The molecular structure and crystallinity of the phytosterol and its ester in the o/w emulsion may be the primary determinants of their loss upon storage, according to prior research on the oxidation of β-sitosterol and β-sitosteryl oleate loaded in o/w emulsion [9]. The oxidations of both β-sitosterol and β-sitosteryl oleate in the o/w emulsion followed a first-order kinetic model, with β-sitosteryl oleate deteriorating at a faster rate than native β-sitosterol.

Lipid oxidations in bulk oils, fats, and emulsions have been extensively studied [16]. For the bulk oil, lipid may be prone to oxidation in a homogeneous medium. Nevertheless, the fatty acid-phytosterol ester can self-assemble in bulk oil as a result of hydrophobic interaction and create a range of related colloids, including reverse micelles and lamellar structures. It is therefore still unknown how the phytosterol ester in bulk oil will react to oxidation. When kept in storage, phytosterol fatty acid esters with unsaturated side chains are more likely to oxidize [9]. This study evaluated the oxidative stability of synthetic β-sitosteryl oleate in comparison to β-sitosterol utilizing a bulk oil model system and accelerated storage conditions (65 °C) for 35 days. This study also focused on the connection between structure and oxidative stability as it relates to oxidation rate.

## 2. Results and Discussion

### 2.1. HPV, TBARS and AnV

**Figure 1:**
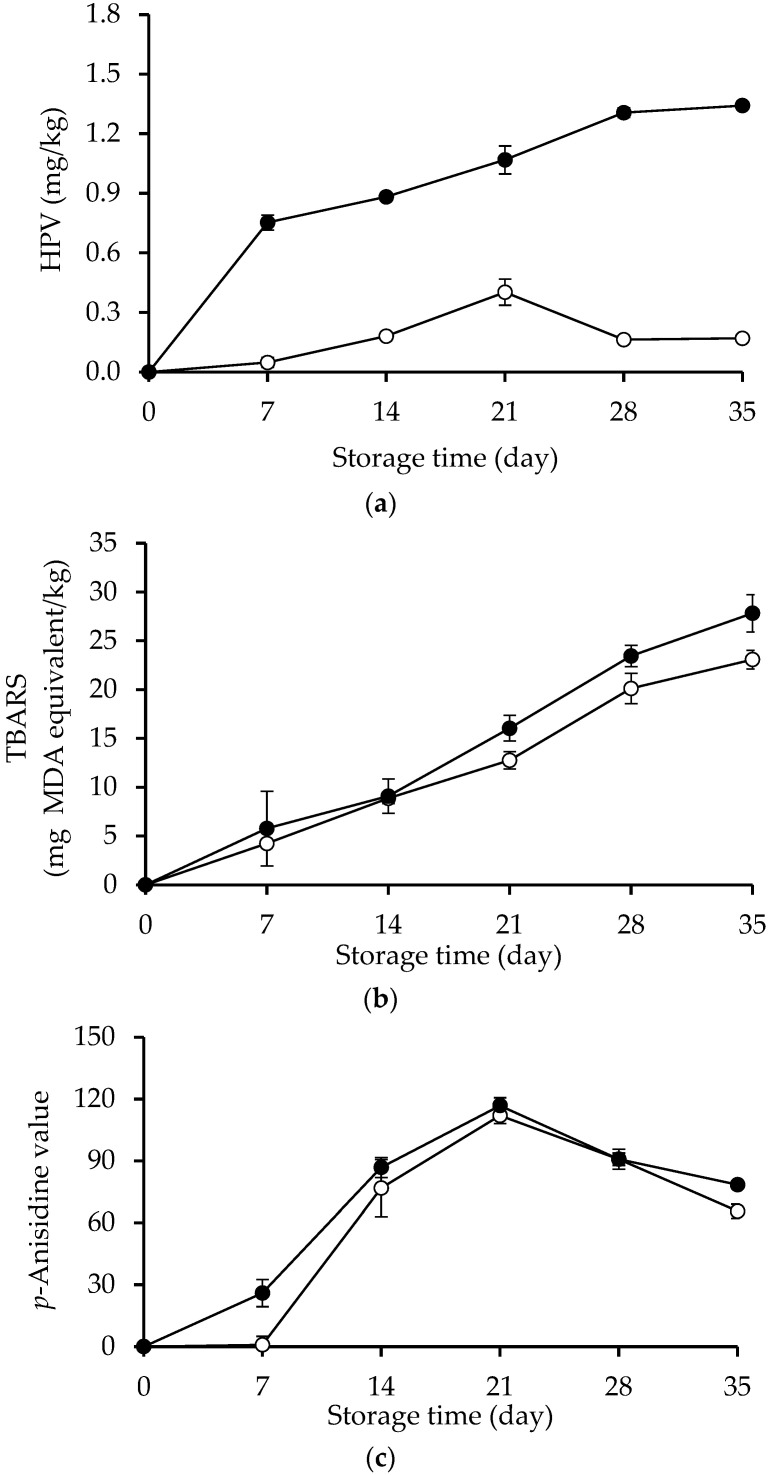
Hydroperoxide value (HPV) (**a**), thiobarbituric acid reactive substances (TBARS) (**b**), and *p*-Anisidine value (AnV) (**c**) of β-sitosterol (o) and β-sitosteryl oleate (•) during incubation at 65 °C for 35 days in bulk oil model system. Error bars represent standard deviations (*n* = 3).

The HPVs of β-sitosteryl oleate and β-sitosterol during incubation (65 °C/35 days) in bulk oil are given in Figure 1a. The main products of β-sitosterol oxidation are generally sitosterol oxides [17]. The oxidation of steryl esters in the lipid matrix has been observed using the peroxide value [18,19,20] and peroxide value reflected total hydroperoxyl groups presented in the sample [21]. Thus, HPV can be used to determine the oxidation level of β-sitosterol and its ester. From the results, it can be postulated that the structure of β-sitosterol conjugated with unsaturated fatty acid led to a higher development of HPV than the unesterified β-sitosterol in bulk oil. Additionally, increasing incubation time caused continuous increase in the HPV of β-sitosteryl oleate (*p* < 0.05), whereas the HPV of native β-sitosterol tended to gradually increase up to 21 days and declined thereafter. The level of β-sitosterol and its ester’s hydroperoxide production may be related to how soluble in fat they are. According to the report, an aliphatic acyl group of oleic acid was deposited into β-sitosterol upon esterification, leading to a significant reduction in the melting point of the product [22]. The melting points of native β-sitosterol and β-sitosteryl oleate were found at 134.1 and 58.5 °C, respectively [22]. Compared to β-sitosterol, β-sitosteryl oleate should be more fat-soluble, and it is also more prone to oxidize quickly in bulk oil.

TBARS of β-sitosterol and β-sitosteryl oleate during an accelerated test in bulk oil (65 °C/35 days) are shown in Figure 1b. TBARS significantly increased in both samples over the course of storage (*p* < 0.05). During the first 14 days, differences in TBARS among samples were not statistically significant (*p* > 0.05). With increasing storage time, β-sitosteryl oleate showed higher TBARS values than native β-sitosterol. Normally, the decomposition of hydroperoxides led to the accumulation of aldehyde species that can be evaluated by TBARS assay. The maximum TBARS value in the bulk oil system was found to be 23–28 mg MDA equivalent/kg at the final stage of the accelerated storage. Cercaci et al. [19] reported that headspace hexanal concentration in bulk corn oil (contained 6481 ± 220 mg phytosterol per kg oil) increased with increasing storage time up to 40 days at 55 °C.

The AnV of the treatments with β-sitosterol and β-sitosteryl oleate upon storage at 65 °C increased with increasing storage time (Figure 1c). The AnV of both treatments increased up to day 21 and dropped thereafter (*p* < 0.05). Native β-sitosterol tended to have a lower AnV. Nieminen et al. [23] used AnV to determine the oxidation levels of spreads supplemented with plant stanol ester throughout storage at 6 °C and the outcome was very different from this research. With chilling, negligible change in the AnV of spreads was observed during 22 days of storage. In this investigation, the AnV grew more quickly at a higher temperature and different matrix.

### 2.2. Concentration of 7-Ketositosterol

**Figure 2:**
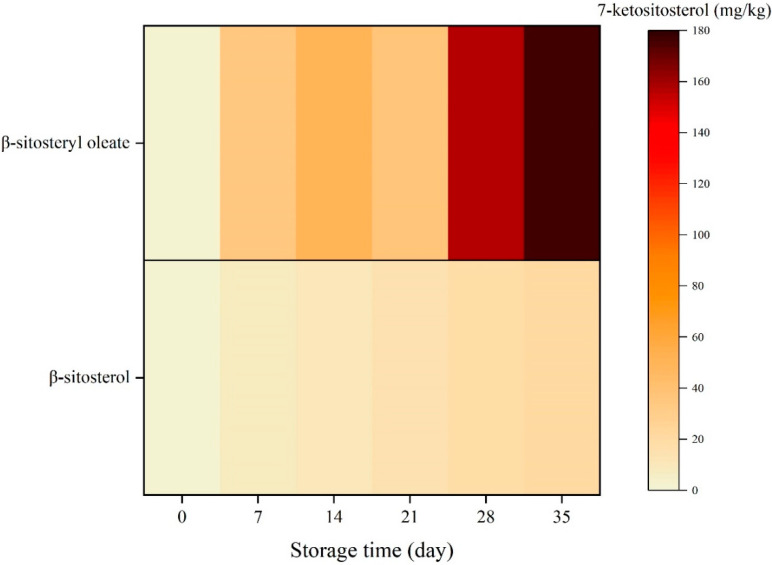
Heat map represents the 7-ketositosterol content of *β*-sitosterol and *β*-sitosteryl oleate during incubation at 65 °C for 35 days in bulk oil model system.

Sterol hydroperoxides generally formed at the allylic C-7 position during oxidation of phytosterol [11], and POPs including keto-derivatives can also be found [12]. The 7-ketositosterol distributions for β-sitosterol and β-sitosteryl oleate are shown in Figure 2.

The amount of 7-keto derivatives in both samples increased for the duration of storage. The outcome was consistent with the findings of Cercaci et al. [19]. However, higher 7-keto derivatives were present in the sample comprising β-sitosteryl oleate than β-sitosterol (*p* < 0.05). In an emulsion model system containing β-sitosterol and β-sitosteryl oleate, the same tendency was observed [9]. This result suggested the strong effect of unsaturated structure on the oxidative deterioration of phytosterol ester.

### 2.3. Remaining of β-Sitosterol and β-Sitosteryl Oleate and Kinetics Evaluation

**Figure 3:**
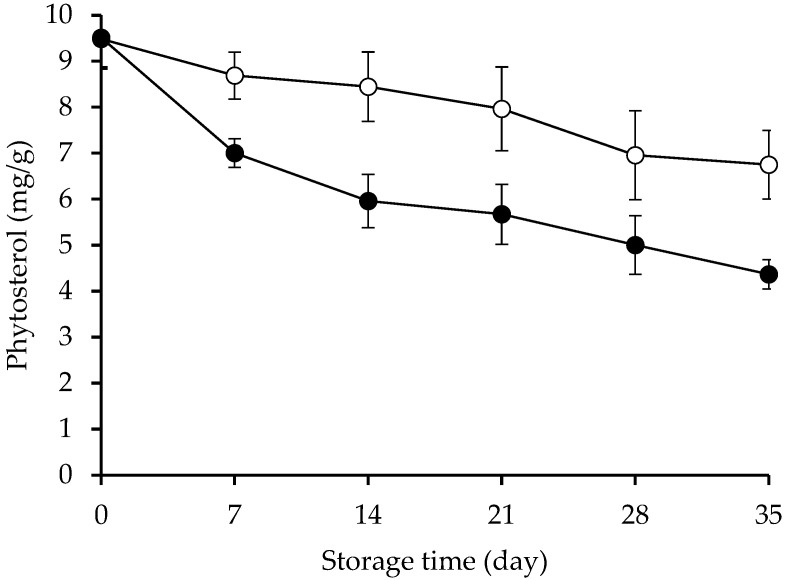
Remaining amount of β-sitosterol (o) and β-sitosteryl oleate (•) during incubation at 65 °C for 35 days in bulk oil model system. Error bars represent standard deviations (*n* = 3).

The remaining amount of β-sitosterol and β-sitosteryl oleate decreased during storage in bulk oil (Figure 3) (*p* < 0.05). The result was consistent with Cercaci et al. [19], who also noticed the decrease in phytosterol content during storage. The remaining levels of -sitosterol and -sitosteryl oleate steadily dropped throughout the course of storage, with -sitosteryl oleate experiencing a greater loss (*p* < 0.05), according to the findings. As a result, in bulk oil, β-sitosteryl oleate oxidized more quickly than β-sitosterol. This was most likely caused by the oxidation of the oleyl size chain and the autooxidation of the phytosterol portion. The physical structure and lipid-solubility can be the factors determining the oxidative stability during storage of phytosterol. The β-sitosteryl oleate may solubilize in oil and some of the native β-sitosterol may precipitate due to its poor solubility in fat [22]. The crystalline nature of β-sitosterol may retard the oxidation, whereas the lipid-solubilized β-sitosteryl oleate may facilitate the oxidation. Overall, the remaining β-sitosteryl oleate and β-sitosterol contents could be governed by the degree and stage of oxidation.

A kinetic evaluation of the reduction of β-sitosteryl oleate and β-sitosterol upon storage was performed (Table 1). All changes in the oxidative indices among samples during the storage fit well with a first order kinetic model (R^2^ = 0.9391–0.9648). The findings were consistent with those made in reports by Ansorena et al. [24] and Panpipat et al. [20], who discovered first order kinetics for the thermo-oxidation of cholesterol and the oxidation of margarine enriched with various structures of β-sitosterol-fatty acid esters throughout storage, respectively. From the results, β-sitosterol showed a lower decreasing rate than β-sitosteryl oleate (Table 1). It was discovered that β-sitosterol (k = 0.0099 day^−1^) was more persistent than β-sitosteryl oleate (k = 0.0202 day^−1^). According to the results, the oxidation and other reactions of β-sitosteryl oleate continuously proceed in bulk oil during storage. Besides the oxidized phytosterols, other products such as oligomers, volatile compounds, and fragmented phytosterol molecules were also detected during thermal destruction [25,26]. This contributed to the decrease in the amount of remaining β-sitosterol and its ester throughout the storage.

## 3. Materials and Methods

### 3.1. β-Sitosterol and β-Sitosteryl Oleate Samples

**Figure 4:**
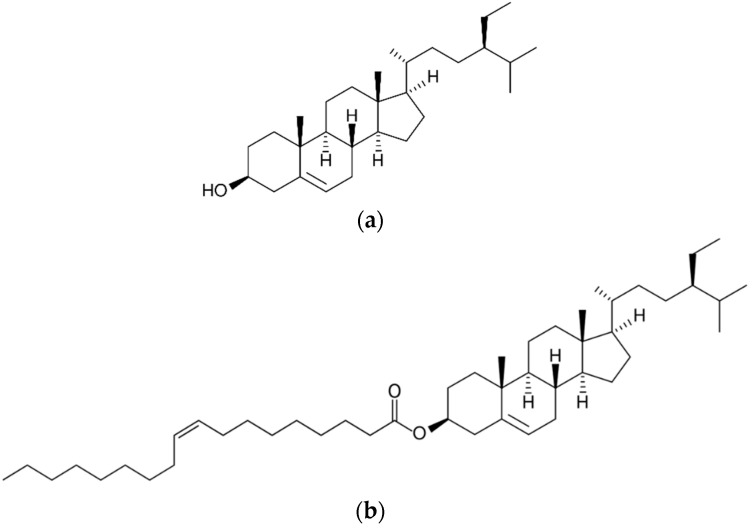
Chemical structures of β-sitosterol (**a**) and β-sitosteryl oleate (**b**).

Since β-sitosterol is the most prevalent phytosterol naturally occurring in plants and plant products [9,20], it was chosen as a model substrate for the production of phytosterol fatty acid ester. Here, synthetic β-sitosterol (purity ≥ 95%) (Figure 4a) was obtained from Sigma-Aldrich (St. Louis, MO, USA). β-sitosteryl oleate (Figure 4b) was enzymatically prepared by the method of Panpipat et al. [22] using immobilized *Candida antarctica* lipase A (CAL A, NZL-101; Codexis, Inc., Pasadena, CA, USA) as a green biocatalyst. In the reaction, β-sitosterol was combined with oleic acid (purity ≥ 95%) at a mole ratio of 1.0:1.0 (mol/mol) at the concentration of 0.2 M in 3 mL of hexane in the presence of 5% CAL A (w% of β-sitosterol) in a 10 mL sealed vial (40 °C/24 h/agitation at 500 rpm). Then, the enzyme was filtered out to terminate the reaction. A Merck-Hitachi HPLC 7100 system (Merck, Darmstadt, Germany) equipped with a silica 60-column and an evaporative light scattering detector (ELSD) was used to track the reaction at 40 °C, using nitrogen as a nebulizing gas at a pressure of 2.2 bar and gain 6. The mobile phases’ elusion program was suggested by a prior report of Panpipat et al. [22]. To purify the end products, the reaction mixtures were run using thin layer chromatography (TLC) on silica gel (silica gel 60 F254; E. Merck Co., Darmstadt, Germany). Cyclohexane/ethyl acetate was used to develop the plates (4:1, *v*:*v*). The band of β-sitosteryl oleate was scraped, collected, and extracted by diethyl ether. After the diethyl ester was evaporated, the β-sitosteryl oleate was obtained and submitted to structural identification. The suggested method provided the β-sitosteryl oleate with a purity higher than 99% according to FTIR and ^1^H NMR results [22].

### 3.2. Oxidation in Bulk Oil Model System

Tripalmitin (purity ≥ 85%), as the mimetic matrix for bulk oil, was supplemented with β-sitosteryl oleate or β-sitosterol at a proportion of 1% (*w*/*w*). To reduce the oxidation of the lipid matrix during storage, tripalmitin was utilized. To simulate the accelerated lipid oxidation system and assure complete solubilization of the matrix, the enriched samples (5.0 g) were exposed to oxidation in sealed 15 mL-glass vials in a Memmert oven (Schwabach, Germany) at 65 °C for 0, 7, 14, 21, 28, and 35 days. At the end of each corresponding experimental run, samples were subjected to analyze for lipid oxidation. Non-oxidized phytosterol/phytosterol ester contents and their reaction rate constant were determined according to Panpipat et al. [20]. To determine the actual oxidation indices of the phytosterol/phytosterol ester, the lipid matrix alone was also tested and used for subtraction from the results of the treatments.

### 3.3. Determination of HPV, TBARS and AnV

HPV, TBARS, and AnV assays were carried out spectrophotometrically based on the standard method of Shantha and Decker [27], Ke and Woyewoda [28], and IUPAC [29], respectively.

### 3.4. Determination of Phytosterol Oxide and Kinetic Evaluation for Sterol Remaining

Phytosterol oxide was determined using the method of Soupas et al. [30] with some modifications as suggested by Panpipat et al. [20] using 19-hydroxycholesterol as an internal standard. The approach of Lampi et al. [31] was used to assess sterol remaining and calculated according to Panpipat et al. [20] as follows:

(1) $$\mathrm{Sterol}\;\mathrm{remaining}\;\left(\%\right)=\frac{\mathrm{Sterol}\;\mathrm{content}\;\mathrm{at}\;\mathrm{time}\;\mathrm{interval}}{\mathrm{Initial}\;\mathrm{sterol}\;\mathrm{content}}\times 100$$

The prior report by Chaijan and Panpipat [9] has information on all approaches.

The first order reaction’s kinetic model was created using the Equation (2).

(2) $$\ln\left(\frac{S_{t}}{S_{0}}\right)=-\mathrm{kt}$$

The sterol contents at time t and time 0 are represented by the symbols S_t_ and S_0_, respectively.

### 3.5. Statistical Analysis

Analysis of variance (ANOVA) was conducted on data, and the treatment means were compared by Duncan’s multiple range test to state the significant differences (*p* < 0.05). SPSS software (10.0, SPSS Inc., Chicago, IL, USA) was applied for statistical analysis. All experiments were triplicated in a completely randomized design.

## 4. Conclusions

The oxidation of phytosterol fatty acid ester with unsaturated side chain was compared to the native phytosterol in the bulk oil model system at accelerated storage (65 °C) in this study. It was found that all oxidation indicators tended to increase throughout storage. The pattern of each index’s evolution, nevertheless, varied according on the sterol structure. Reduction of β-sitosterol and its ester was fitted to a first order kinetic model. It appeared that β-sitosteryl oleate with an unsaturated side chain oxidized more quickly than natural β-sitosterol during storage. Therefore, the storage stability against oxidation of phytosterol in bulk oil was mainly governed by its physical structure. The results can be used as a guideline for designing the strategies to prevent oxidative loss of β-sitosteryl oleate containing fat and oil products during storage.

## Figures and Tables

**Table 1:** The reaction rate constant of remaining β-sitosterol and β-sitosteryl oleate after 35 days of incubation at 65 °C in a model system using bulk oil.

β-Sitosterol Species	Reaction Rate Constant (k; day^−1^)	R^2^
Unesterified β-sitosterol	0.0099	0.9648
β-sitosteryl oleate	0.0202	0.9391

Model of first order reaction according to $\ln\left(\frac{S_{t}}{S_{0}}\right)=-\mathrm{kt}.$

## Data Availability

Not applicable.

## Sample Availability

Samples of the compounds are available from the authors.
